# Studying the Effect of Four Postspace Preparation Techniques on the Apical Seal Integrity of the Root Canals Obturated With the Resilon/RealSeal System and the Lateral Condensation Technique

**DOI:** 10.1155/ijod/6635157

**Published:** 2025-09-11

**Authors:** Emad Elsubeihi, Lina Haddadien, Mohamed Elsayed

**Affiliations:** ^1^College of Dentistry, Ajman University, Ajman P.O. Box 346, UAE; ^2^Center of Medical and Bio-allied Health Sciences Research, Ajman University, Ajman P.O. Box 346, UAE; ^3^Endodontic Department, Faculty of Dentistry, Mansoura University, Mansoura, P.O. Box 35516, Egypt

## Abstract

**Objectives:** The objective of this study was to investigate the effect of different postspace preparation techniques on the apical seal of Resilon/RealSeal obturation system in root canals filled with the lateral condensation technique.

**Methods:** A total of 92 maxillary central incisors were selected for this study, and their crowns were cut. The root canals were instrumented using a Reciproc file system and then filled with a Resilon/RealSeal obturation system, using the lateral condensation technique. Seventy-five samples were divided into five groups (*n* = 15) according to postspace preparation techniques: Group 1 (no postspace preparation), Group 2 (immediate/mechanical postspace preparation), Group 3 (immediate/thermal postspace preparation), Group 4 (delayed/mechanical postspace preparation), and Group 5 (delayed/thermal postspace preparation). The remaining 16 roots were allocated as follows: 10 roots were used as control groups, while seven roots were used for the preparation of blank solutions. The effectiveness of the apical seal of the obturation material was evaluated using the dye extraction method. The absorbance values of the extracted dye in different groups were measured using a spectrophotometer, and their concentrations (µg/mL) were calculated based on a preestablished calibration curve. Data were statistically analyzed for comparison among groups.

**Results:** All experimental groups showed significant differences in apical leakage (*p* < 0.05). The lowest mean dye leakage was observed in Group 1 (0.127 ± 0.041 µg/mL) and Group 3 (0.174 ± 0.076 µg/mL). However, no significant difference was found between these two groups (*p*  > 0.05). Group 3 demonstrated significantly lower leakage compared to Groups 2, 4, and 5 (*p*  < 0.05).

**Conclusions:** The Resilon/RealSeal system used with the lateral condensation technique demonstrated a reduction in apical dye leakage but failed to completely prevent it, especially when postspace preparation was not performed. Among the evaluated techniques, immediate/thermal postspace preparation proved to be the most effective in maintaining the integrity of the apical seal. In contrast, delayed/thermal, delayed/mechanical, and immediate/mechanical techniques significantly compromised the apical seal. Selecting an optimal postspace preparation technique is essential to preserve the apical seal and enhance the long-term success of endodontic treatments.

## 1. Introduction

A three-dimensional root canal filling is essential to prevent apical and coronal leakage within the root canal system [1]. Posts are often required in endodontically treated teeth to retain a core material that restores the lost coronal tooth structure [2]. However, the creation of a postspace is a critical step, as it necessitates the removal of the coronal portion of the root canal filling material, which can compromise the apical seal of the remaining filling [3].

Gutta-percha (GP) is universally recognized as the gold standard material for root canal filling [4]; however, advancements in dentin bonding have led to the development of resin-based root canal filling material, such as the RealSeal system, which claims to bond to root dentin and create a monoblock structure. RealSeal (Resilon) was introduced in 2004 and is composed of a parent polymer, polycaprolactone, which is a biodegradable aliphatic polyester, reinforced with filler particles, including bioactive glass, bismuth oxychloride, and barium sulfate [5]. The accompanying sealer, RealSeal, originally named Epiphany, is a dual-curable resin-based composite sealer. Its matrix is a mixture of bisphenol A glycidyl methacrylate (Bis-GMA), ethoxylated (Bis-GMA), urethane dimethacrylate (UDMA), and hydrophilic difunctional methacrylate, with fillers such as calcium hydroxide, barium sulfate, barium glass, and silica [6]. The literature supports the use of RealSeal and similar materials, with numerous studies reporting reduced microleakage rates compared to traditional root canal filling systems [7, 8]. Additionally, some studies suggest that these materials increase the fracture resistance of endodontically treated roots [9, 10].

Postspace preparation can be done immediately after root canal obturation or in a delayed stage. Several studies have highlighted the benefits of immediate postspace preparation, particularly in minimizing microleakage when AH Plus sealer is used with GP to fill the root canals [11–13]. Al-Ashou et al. [12] reported significantly reduced microleakage in roots filled with GP in combination with resin-based or bioceramic sealers when postspace preparation was performed immediately. Furthermore, contrarily, Dhaded et al. [14] found that delayed postspace preparation in teeth obturated with Resilon/RealSeal resulted in superior wall adaptation and lower microleakage compared to GP combined with AH Plus.

Different methods have been proposed for removing root canal filling materials during postspace preparation, including chemical, mechanical, and thermal techniques. Among these, chemical methods are associated with a higher risk of microleakage [15]. However, the impact of thermal vs. mechanical removal techniques on apical leakage remains controversial. While some studies have not found significant differences between these two methods [16, 17], others have demonstrated that thermal removal results in less apical leakage compared to mechanical techniques during postspace preparation [15]. Wagnild and Mueller [18] suggested that the thermal removal of GP should be the preferred method, reserving mechanical techniques for cases where thermal methods are insufficient. Despite this, there is a notable gap in the literature regarding the comparative effects of immediate vs. delayed postspace preparation using either thermal or mechanical methods on apical leakage in root canals filled with the Resilon/RealSeal system.

Sealing ability is an essential characteristic that must be tested for all root canal filling materials or techniques. Dye penetration techniques are the most commonly used methods to evaluate the sealing ability of dental materials due to their simplicity and efficiency [19]. Among these, the dye extraction method provides superior reliability, since it quantitatively measures the total uptake of dye within the root [20, 21].

Therefore, the objective of this study was to use the dye extraction method to investigate the effect of different postspace preparation techniques (immediate/mechanical, immediate/thermal, delayed/mechanical, and delayed/thermal) on apical leakage in root canals obturated with the Resilon/RealSeal system using the lateral condensation technique. The null hypothesis was that there are no differences in apical leakage values among the different postspace preparation techniques.

## 2. Materials and Methods

This in vitro study was conducted using caries-free extracted human maxillary central incisors and received approval from the local Research Ethics Committee (RD-2014/15-21) for the ethical use of biological materials.

Informed consent was obtained from all patients for the use of their extracted teeth in this study. The teeth were collected from dental clinics at Ajman University, UAE, as well as from private dental practices in Ajman, Sharjah, and Dubai. Immediately after extraction, the teeth were rinsed thoroughly with water and sterilized by autoclaving at 121°C under 15 psi pressure for 40 min, following the protocol described by Kohn et al. [22]. Subsequently, soft tissue remnants and calculus were mechanically removed [22] and the teeth were then stored in 0.1% thymol solution at 4°C until use.

### 2.1. Sample Selection and Preparation

A sample size calculation was performed using G^*⁣*^*∗*^^Power software (version 3.1.9.7, Heinrich-Heine-Universität Düsseldorf) and data obtained from a preliminary pilot study, with *α* = 0.05, power = 0.80, and an effect size of 0.40, resulting in a minimum of 15 samples per group.

An additional 17 teeth were included to prepare control samples and blank solutions.

All teeth were screened visually, microscopically, and radiographically to confirm suitability. Exclusion criteria included previously treated teeth, defective roots, severely curved or calcified canals, and open apices.

Each tooth was decoronated at the cementoenamel junction using a diamond disc (Edenta, Switzerland) under continuous water irrigation. Roots exceeding the desired length were trimmed as needed to standardize their length to 15 mm.

### 2.2. Root Canal Preparation

The working length was established by inserting a 10K-file (Technical & General Ltd., London, UK) into the canal until it was visible at the apical foramen, then subtracting 1 mm from this measurement. Root canals were cleaned and shaped using a Reciproc NiTi rotary file size R50 (VDW GmbH, Munich, Germany) in accordance with the manufacturer's instructions. After each file, canals were irrigated with 5 mL of 1% sodium hypochlorite (NaOCl) solution delivered through a 27-gauge Max-i-Probe needle (Dentsply Maillefer, Ballaigues, Switzerland).

Upon completion of preparation, apical foramen patency was confirmed with a 10K-file. Final irrigation consisted of 5 mL of 1% NaOCl followed by 3 mL of 17% EDTA solution (Meta Biomed Co. Ltd., United Kingdom) for 1 min to remove the smear layer. A final flush of 10 mL distilled water ensured removal of any residual NaOCl or EDTA. The canals were dried with Reciproc paper points of size 50 (VDW GmbH, Munich, Germany) until a completely dry paper point was obtained.

### 2.3. Study Groups

The filled roots (*n* = 75) were randomly divided into five groups of 15 samples each, according to the postspace preparation technique: Group 1 (Control group): no postspace preparation; Group 2: immediate/mechanical postspace preparation; Group 3: immediate/thermal postspace preparation; Group 4: delayed/mechanical postspace preparation; Group 5: delayed/thermal postspace preparation. Randomization was performed using an online software tool (https://www.randomizer.org/), operated by a blinded individual to ensure allocation concealment and eliminate bias. The remaining samples (*n* = 17) were distributed as follows: five samples each served as positive and negative controls [23], and seven samples were used to prepare blank solutions for spectrophotometer calibration during the leakage assessment (one blank solution per group).

### 2.4. Application of Nail Varnish to the Samples

After finishing root canal preparation, the outer surface of each root in the experimental and positive control groups was coated with three layers of clear nail varnish (Lancome, France), leaving the apical foramen exposed. To prevent the nail varnish from entering the apical foramen, a size 10 spreader (Dentsply) was inserted into the canal through the apical foramen until it reached the maximum diameter of the foramen. Each varnish layer was allowed to dry fully before applying the subsequent layer. For the negative control group, all root surfaces, including the apical foramina, were entirely coated with three layers of nail varnish. In contrast, the samples designated for blank solution preparation were not coated with nail varnish. After the coating process, each root was wrapped in gauze moistened with distilled water, placed in labeled plastic containers, and stored in an incubator set at 37°C with 100% humidity until further use. This ensured consistent environmental conditions for all samples.

### 2.5. Root Canal Obturation

The lateral condensation technique was selected to obturate the root canals with the RealSeal system (RealSeal, SybronEndo, Orange, CA, USA). The procedure began with the application of RealSeal primer using a fully dampened paper point inserted to the full working length of each canal. Excess primer was removed with a size 50/0.02 paper point (Dentsply Tulsa Dental Specialties). RealSeal sealer was then prepared using the manufacturer's specialized spiral tip and introduced into the canal with a lentulo spiral (Dentsply Maillefer, USA) to ensure uniform coating of the canal walls. A Resilon master cone (size 50/0.02), coated with RealSeal sealer, was inserted to the full working length. Lateral condensation was performed using a finger spreader and fine–fine accessory Resilon cones (RealSeal, SybronEndo, Orange, CA, USA) until the canal was completely filled.

**Figure 1:**
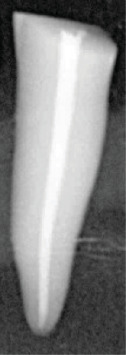
Periapical radiograph demonstrating a root canal system filled with obturation material, indicating adequate canal filling and adaptation.

Excess Resilon material at the coronal orifice was removed with a heated instrument, and the coronal portion was light-cured for 40 s using an LED curing unit (Lite 696, Dentamerica, USA). Radiographs were taken to verify the quality of obturation (Figure 1), and samples exhibiting internal voids were replaced with new ones. The control group samples were obturated following the same procedure as the experimental groups but without the application of sealer. Similarly, samples designated for blank solution preparation (one sample per group) underwent the same obturation and postspace preparation protocols as the experimental and control groups.

### 2.6. Postspace Preparation

In the immediate postspace preparation protocol, roots in Groups 2 and 3 were incubated at 37°C for 30 min. Postspace preparation was then performed by removing the coronal 10 mm of filling material either mechanically (Group 2) or thermally (Group 3). For delayed postspace preparation, roots in Groups 4 and 5 were incubated at 37°C for 7 days before removing the coronal 10 mm of filling material mechanically (Group 4) or thermally (Group 5).

In Groups 2 and 4, mechanical removal of the filling material was accomplished using a Gates Glidden drill size 3 (Technical & General Ltd., London, UK). In Groups 3 and 5, thermal removal was performed with a Buchanan electric heat plugger size 1 (Fine-50/0.6 taper) attached to the Elements Free System (Kerr Corporation, 1717 West Collins, Orange, CA, USA). The device temperature was set at 150°C to ensure effective thermal removal of the filling material.

### 2.7. Mechanical Postspace Preparation After Root Canal Filling Removal

**Figure 2:**
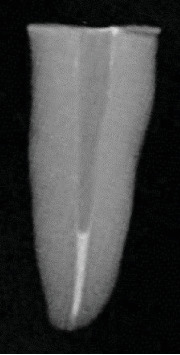
Periapical radiograph illustrating the apical seal following postspace preparation.

After the coronal 10 mm of root canal filling material was removed, either mechanically or thermally, the postspace was mechanically widened using a Peeso reamer size 3 (Mani, Tochigi, Japan). The reamer was advanced into the canal until it reached 0.5 mm short of the apical end of the remaining GP to preserve the integrity of the apical seal. Debris generated during the mechanical postspace preparation was removed using a curved luer lock suction tip, and a directed air stream was applied to clean the postspace thoroughly. Radiographs were taken for all samples to confirm the length of the remaining root canal filling material and ensure procedural accuracy (Figure 2).

Subsequent to postspace preparation, the coronal access cavity of each root was sealed using light-cured glass ionomer cement (FX-II capsule, Shofu Inc., Japan). The coronal portion of the root, including the glass ionomer filling, was coated with three layers of clear nail varnish to prevent dye penetration. Samples assigned to the immediate postspace preparation protocol were incubated for 1 week at 37°C with 100% humidity to ensure complete sealer setting.

### 2.8. Apical Microleakage Assessment

#### 2.8.1. Application of Dye Solution

**Figure 3:**
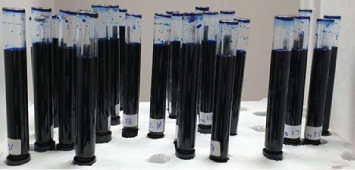
Arrangement of glass test tubes within perforations of an expanded polystyrene (thermocol) container.

The dye extraction technique, as described by Camps and Pashley [24], was employed to quantify dye leakage in µg/mL. A 2% aqueous solution of methylene blue (MB) dye was used to evaluate the apical seal of the obturated canals with and without postspace preparation. For the dye leakage test, each root sample was placed through a standardized central hole drilled into the plastic cap of a glass test tube, ensuring that only 1 mm of the apical root extended into the test tube's interior. The cap was sealed with wax to secure the sample in place. The test tubes were filled with 2% MB dye solution up to a premarked 7 cm level. The assembly was then vertically positioned in holes made in a thermocol box (Figure 3), ensuring that the root apex faced upward and was fully submerged in the dye solution. To prevent light exposure, the test tubes were wrapped in aluminum foil and incubated for 72 h. The dye level was monitored daily, and any decrease in volume was replenished with fresh dye to maintain consistency throughout the storage period. After the incubation period, the roots were removed from the test tubes and rinsed under running water for 30 min to eliminate excess dye. Nail varnish coatings were removed using a #15 Parker blade and polishing discs, while the coronal glass ionomer filling was carefully removed with a size 1 low-speed carbide round bur (Jota, Switzerland).

#### 2.8.2. Method of Dye Extraction

The dye extraction method involved immersing each root sample in a hermetically sealed glass test tube containing 4 mL of 65% nitric acid (Emsure, Germany) for 3 days to dissolve the MB dye within the root canals. After the immersion period, the test tubes were centrifuged at 4000 rpm for 5 min to precipitate filling and dentin debris. The resulting solution was further filtered through glass wool (Surechem, UK) to remove residual particulate matter.

**Figure 4:**
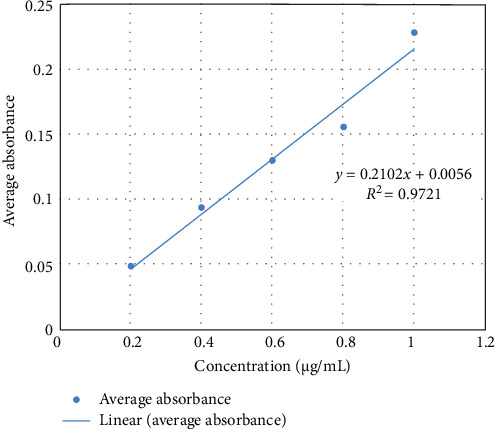
Calibration curve of methylene blue showing absorbance as a function of concentration (µg/mL).

Serial dilutions of MB dye were prepared to generate standard concentrations ranging from 1 to 0.2 μg/mL. Absorbance values for these standards were measured at a wavelength of 584 nm using a spectrophotometer (Shimadzu UV-1900, Maryland, USA). A calibration curve was constructed in Microsoft Excel, demonstrating a linear relationship between dye concentration and absorbance. The calibration curve equation (Figure 4) was *Y* = 0.0117*X* − 0.0014*Y*, where *Y* represents absorbance and *X* represents dye concentration in μg/mL.

To ensure measurement accuracy, the nitric acid solution obtained from dissolving untreated roots was used as a blank for spectrophotometer calibration, eliminating any interference from background absorbance. Absorbance values for the experimental samples were then recorded and converted into dye concentrations using the linear regression equation derived from the calibration curve. Each sample measurement was performed in triplicate, and mean values were calculated to ensure precision. The concentration of extracted dye, expressed in μg/mL, served as an indicator of apical leakage, enabling an accurate assessment of the sealing efficacy of the tested materials. This rigorous approach ensured reliable and reproducible quantification of dye penetration, offering valuable insights into the performance of different root canal obturation techniques.

### 2.9. Statistical Analysis

The normality of data distribution was confirmed using the Shapiro–Wilk test (*p*  > 0.05), indicating a normal distribution. Homogeneity of variances across groups was verified with Levene's test. As the assumptions for parametric analysis were met, one-way analysis of variance (ANOVA) was employed, followed by Tukey's post hoc test for multiple comparisons. Statistical analyses were performed using IBM SPSS Statistics, version 20 (IBM Corp., Armonk, NY, USA). A *p*-value < 0.05 was considered statistically significant.

## 3. Results

**Figure 5:**
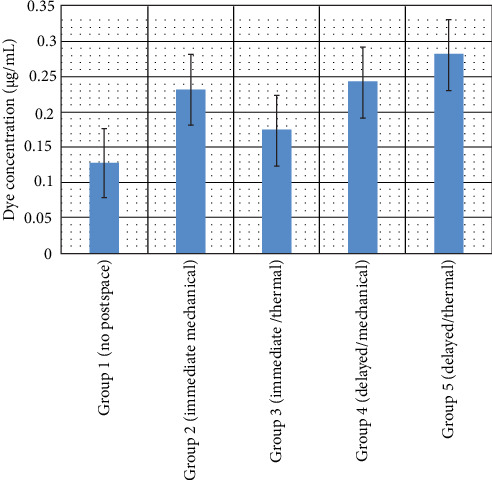
Graphical representation of mean methylene blue concentrations (µg/mL) across all experimental groups.

The mean concentrations and standard deviations of the extracted MB dye (µg/mL) for all experimental groups are summarized in Table 1 and illustrated in Figure 5. All experimental groups exhibited some degree of apical dye leakage, though the extent varied depending on the timing and method of post space preparation. One-way ANOVA revealed statistically significant differences among the groups (*p*  < 0.05), indicating that the post space preparation technique and timing had a measurable effect on apical seal integrity.

Group 1 (no post space preparation) demonstrated the lowest mean dye concentration, indicating the most effective apical seal among the experimental conditions. Group 3, which underwent immediate thermal post space preparation, exhibited a similarly low dye concentration, with no statistically significant difference from Group 1 (*p*  > 0.05). This suggests that immediate thermal preparation preserved the integrity of the apical seal effectively.

In contrast, Groups 2 (immediate mechanical), 4 (delayed thermal), and 5 (delayed mechanical) showed significantly higher mean dye concentrations compared to Groups 1 and 3 (*p*  < 0.05), reflecting compromised apical seals. Among these, Group 5 demonstrated the highest leakage, with a mean dye concentration of 0.281 ± 0.166 µg/mL, highlighting that delayed mechanical preparation posed the greatest risk of disrupting the apical seal. However, there were no statistically significant differences in leakage among Groups 2, 4, and 5 themselves (*p*  > 0.05), indicating a comparable degree of apical compromise across these conditions.

The control groups further validated the experimental methodology. The positive control group, with no apical seal, displayed the highest dye penetration (mean: 5.89 µg/mL), confirming complete leakage. In contrast, the negative control group, in which the apical foramen was completely sealed with nail varnish, exhibited minimal dye penetration (mean: 0.011 µg/mL), confirming the reliability and specificity of the dye extraction method for detecting apical leakage.

## 4. Discussion

Apical leakage is a critical factor contributing to endodontic treatment failure. It is influenced by multiple variables, including the obturation technique, physicochemical properties of the sealer, and smear layer removal [25]. The development of resin-based root canal filling systems, such as Resilon/RealSeal, aimed to improve adhesion to radicular dentin and create a monoblock structure to enhance the apical seal, reduce leakage, and increase resistance to root fracture [9, 10]. Despite their theoretical advantages, the clinical performance of these systems compared to traditional GP remains controversial. Some studies have reported increased leakage with Resilon/RealSeal [8, 26], whereas others observed reduced apical and coronal leakage, especially in short-term evaluations [27, 28].

The timing of postspace preparation also plays a significant role in maintaining the integrity of the apical seal. Conflicting evidence exists in the literature: some studies suggest that postspace preparation timing does not significantly affect leakage [29, 30], while others support immediate preparation due to lower microleakage [31, 32]. In contrast, a few researchers observed less leakage with delayed preparation in GP-filled canals [13, 33]. However, a broader literature review concluded that delayed preparation could compromise apical sealing and recommended immediate preparation as a more reliable clinical approach [33].

The method used for postspace preparation, mechanical vs. thermal, further impacts sealing ability. Thermal removal techniques, such as hot pluggers or systems like Elements Free, have shown better outcomes compared to mechanical approaches like Gates Glidden drills [34, 35]. However, there is a lack of data on the influence of both timing and technique when Resilon/RealSeal is used in combination with the lateral condensation technique, which is still considered a gold standard for obturation [36, 37]. This study aimed to address this gap.

Standardized protocols were followed to reduce anatomical and procedural variability. Maxillary incisors were selected based on apical diameter uniformity. Reciproc NiTi rotary instruments ensured consistent shaping and debris removal [38]. A validated irrigation protocol was used (1% NaOCl, 17% EDTA, and distilled water). While NaOCl may inhibit resin bonding by producing an oxygen-inhibited layer on dentin [39], careful drying with paper points helps mitigate this effect [40].

In our study, four groups were compared based on postspace preparation timing (immediate vs. delayed) and method (mechanical vs. thermal). A 5 mm apical plug of filling material was preserved in all samples [12, 41], as recommended to maintain apical sealing integrity [42, 43]. Postspace preparation was performed using either Gates Glidden drills or the Elements Free system at 150°C, a temperature well below Resilon's degradation point of 350°C [44].

To quantitatively assess leakage, the dye extraction method with MB was used, offering reproducibility and reduced operator bias [24, 45–47]. The dye's small molecular size makes it suitable for leakage evaluation [24, 45, 48], though its affinity for cementum and dentin can pose challenges. To control this, the root surface was coated with nail varnish, leaving only the apical foramen exposed, and blanks were prepared to normalize for intrinsic tooth color [24, 49].

Significant differences in leakage were found among the groups, necessitating the rejection of the null hypothesis. The highest leakage occurred in canals with delayed postspace preparation using the mechanical removal technique. The lowest leakage was shown with immediate postspace preparation using the thermal removal, technique. These results support the clinical recommendation for performing postspace preparation soon after obturation, particularly using thermal methods.

Previous studies have yielded mixed results. While some found higher leakage with delayed postspace preparation in Resilon-filled canals [50, 51], our study found no significant difference between immediate and delayed mechanical groups. Bodrumlu et al. [52] reported reduced leakage with delayed postspace preparation in Resilon/Epiphany-filled roots and less leakage in immediate preparation, though not statistically significant. Discrepancies may stem from variations in irrigation (e.g., use of 5.25% NaOCl vs. 1%) or assessment techniques (fluid filtration vs. dye extraction).

The superior sealing observed in the immediate thermal group may be attributed to the sealer remaining within its working time during preparation, thus avoiding disruption to the bonding interface [31]. Conversely, delayed mechanical preparation likely causes more disruption to the sealer-dentin interface, leading to microcracks or voids [53]. Additionally, thermal removal methods may promote higher resin conversion rates and enhanced flow of the Resilon material due to its thermoplastic nature, contributing to improved adaptation and sealing [54–58].

This study has limitations. It assumes leakage occurs exclusively via the apical foramen, although in vivo, accessory and lateral canals may also serve as leakage pathways. The use of only straight canals does not represent clinical complexities such as curvature and anastomoses. While dye extraction provides quantitative results, it is destructive and does not permit longitudinal assessments. Despite these limitations, in vitro studies offer ethical and practical advantages and remain a valuable tool in evaluating endodontic materials [24, 48, 59].

In conclusion, both the timing and technique of postspace preparation significantly influence apical sealing in canals obturated with Resilon/RealSeal. Immediate postspace preparation, preferably within 30 min of obturation and using a thermal method such as Elements Free, results in significantly less apical leakage. These findings provide evidence-based guidance for clinicians, reinforcing the importance of early postspace preparation with thermally controlled systems to enhance the long-term success of endodontic therapy. Further research using advanced imaging (e.g., micro-CT, CLSM) and long-term aging models is recommended to better simulate clinical scenarios and refine treatment protocols.

## 5. Conclusions

Within the limitations of this in vitro study, the following conclusions can be drawn:

1. The Resilon/RealSeal obturation system, when used with the lateral condensation technique and without postspace preparation, demonstrated the lowest level of apical leakage. However, it did not entirely eliminate leakage.
2. Immediate postspace preparation using thermal technique did not significantly compromise the apical seal of the Resilon/RealSeal system when used with the lateral condensation technique, suggesting it is a clinically favorable approach.
3. Postspace preparation performed using mechanical methods, whether immediate or delayed, as well as delayed thermal preparation, significantly compromised the integrity of the apical seal in canals obturated with Resilon/RealSeal and the lateral condensation technique.

## Figures and Tables

**Table 1:** Comparison between mean values of extracted dye (µg/mL) for all experimental groups.

Groups	Postspace preparation techniques	Concentration of extracted dye (µg/mL)		
		Mean ± SD	Minimum	Maximum
Group 1	No postpreparation	0.127 ± 0.041^a^*⁣*^*∗*^	0.051	0.207
Group 2	Immediate mechanical	0.231 ± 0.083^b^	0.099	0.341
Group 3	Immediate thermal	0.174 ± 0.076^a^	0.053	0.285
Group 4	Delayed mechanical	0.242 ± 0.182^b^	0.104	0.619
Group 5	Delayed thermal	0.281 ± 0.166^b^	0.048	0.631
ANOVA (*p*-value)	—	0.000	—	—

*⁣* ^*∗*^Tukey HSD test: means with different superscript letter in the same column are significantly different (*p*  < 0.05).

## Data Availability

The data used to support the findings of this study will be available from Mohamed Elsayed at this email: elsayednada@yahoo.com for the researchers who meet the criteria for access this data. The data can be requested after the publication of this article. However, requests for the data, 6/12 months after the publication of this article, will be considered by the corresponding authors.
